# Cassava/peanut intercropping improves soil quality via rhizospheric microbes increased available nitrogen contents

**DOI:** 10.1186/s12896-020-00606-1

**Published:** 2020-02-28

**Authors:** Xiumei Tang, Ruichun Zhong, Jing Jiang, Liangqiong He, Zhipeng Huang, Guoying Shi, Haining Wu, Jing Liu, Faqian Xiong, Zhuqiang Han, Ronghua Tang, Longfei He

**Affiliations:** 1 Agricultural College of Guangxi University, Nanning, 530005 Guangxi China; 2 Cash Crops Research Institute, Guangxi Academy of Agricultural Sciences, Nanning, 530007 Guangxi China; 3 Microbiology Research Institute, Guangxi Academy of Agricultural Sciences, Nanning, 530007 Guangxi China

**Keywords:** Cassava, Intercropping, Microbial community, Peanut, Rhizospheric soil

## Abstract

**Background:**

Intercropping, an essential cultivation pattern in modern agricultural systems, increases crop yields and soil quality. Cassava and peanut intercropping systems exhibit advantages in solar utilization and cadmium absorption, etc. However, the inner mechanisms need to be elucidated. In this study, Illumina MiSeq platform was used to reveal the rhizospheric microbes and soil quality in cassava/peanut intercropping systems, and the results provided a reference for the application of this method in studying other intercropping systems.

**Results:**

Both intercropping cassava/peanut (IP) and intercropping peanut/cassava (IC) systems significantly increased available N, available K, pH value, and urease activity, comparing with that in monocropping cassava (MC) and monocropping peanut (MP) system. However, there were few effects on the total N, total P, total K, available P, organic matter, protease activity, catalase activity, sucrase activity, and acid phosphatase activity. Both IP and MP soils contained more bacteria and fungi than those in the IC and MC soils, which were mainly made of Proteobacteria and Actinobacteria. Intercropping remarkably increased the number of Nitrospirae in IP and IC soils comparing those in MC and MP soils. Redundancy analysis (RDA) revealed that the abundances of *DA101*, *Pilimelia*, and *Ramlibacter* were positively correlated to the soil quality. These results suggest that intercropping enhances the available nitrogen content of soil through increasing the quantity of rhizospheric microbes, especially that of *DA101* and *Pilimelia*.

**Conclusions:**

The cassava/peanut intercropping system improves soil quality through increasing the available nitrogen content and abundance of *DA101*, *Pilimelia*, and *Ramlibacter* in the soil.

## Background

Intercropping, an old and effective planting method of growing one crop/animals alongside another, can increase yields, reduce pests [1] and weeds [2], etc. There are many intercropping models, such as chickpea/maize [3], maize/lablab [4], rubber/plantain [5], even marine finfish in shrimp ponds [6]. Intercropping showed many advantages, like higher yield [7, 8], higher light interception and utilization rate [9], phytoremediation of heavy metal contaminated soils [10], enhancing iron nutrition [11] and phosphorus availability [12], etc.

Cassava (*Manihot esculenta* Crantz) is an important food crop in the world [13], especially in Africa [14]. Since its importance, breeding high yield and low cyanide cultivars [14], cultivation [15, 16], genome evolution [17] as well as diseases control were widely studied [18]. Peanut (*Arachis hypogaea* Linn.) is a legume crop that has edible seeds and oils. In cassava, cultivation and nitrogen fixation [19], abiotic stress-responsive ESTs [20], disease resistance genes [21] were well studied. Interestingly, continuous cropping of peanut changes the soil bacterial community [22]. Previous studies showed that peanut/maize intercropping changed rhizosphere and nutrient concentrations in shoots [23], but showed no effects on *Aspergillus flavus* in soil [24].

Recently, the inner mechanisms of intercropping are found related to microorganisms [25]. The progress in molecular [26] and microbiome techniques [27] provided new tools for the elucidation of the mechanisms of intercropping. For instance, molecular mechanisms of microbial disease control in intercropping were related to signals triggered by neighboring plants [28]. Sugarcane-soybean intercropping increased microbial diversity [29]. Microbiome-dependent immunity was related to soil organic matter content [30]. It was reported in previous studies that intercropping can change the soil microecology, as indicated by increasing farmland biodiversity [31]. Intercropping can effectively improve the mobilization and uptake of nitrogen (N), phosphorus (P), potassium (K), and micronutrients via interspecific interactions in the rhizosphere soil [23]. In addition, legume/cereal intercropping systems could improve the utilization of phosphorus (P) by root exudation of organic acids from legume crops which also improve legume N uptake by enhanced nodulation of legume crops [32]. Soil microbe and soil enzyme activities play essential roles in nutrient cycling, organic matter decomposition, and suppression of soil-borne pathogens in the rhizosphere [33, 34]. Plants can release root exudates, thereby affecting the rhizospheric microbial community [35]. Changes can influence the potential activities of soil enzymes in microbial community composition. Due to the quantitative and qualitative differences between the root exudates of intercropping and monocropping systems, differences in the microbial community can be observed [36]. Many studies have investigated the changes in the biochemical and microbial characteristics of rhizospheric soils caused by intercropping [37]. For the alfalfa/rye intercropping system, intercropping affected the soil microbial composition and soil enzymatic activities [38]. Through phospholipid fatty acid (PLFA) analysis, it was found that the soil urease activities, invertase activities, and the soil gram-negative (G-) bacterial abundance were significantly increased in the peanut/*Atractylodes lancea* system [39]. Many studies have shown that the maize/peanut intercropping system can facilitate the acquisition of Fe and Zn by peanut and improve the yields of both crops [23].

Cassava/peanut intercropping is a typical intercropping cultivation mode in southern China. Since the original spacing of the cassava crop remains unchanged when intercropped with peanut, thereby it provides a distinct yield advantage. Although researches were conducted regarding the selection of the cassava/peanut intercropping model and the associated yield benefits, study on the molecular mechanisms underlying this cultivation system remains insufficient. Previous studies on cassava/peanut intercropping mainly focused on the uptake and utilization of nutrients, photosynthesis, agronomic traits, yield, efficiency, and nutrient conversion efficiency in the soil [40]. However, the influence of the microecological soil environment in the cassava/peanut intercropping system remains unknown. In this study, the inner mechanisms of cassava/peanut intercropping were elucidated through the analysis of rhizospheric soil quality and microbial community using the Illumina MiSeq platform. We found that cassava/peanut intercropping enhances the quantity of *DA101* and *Pilimelia*, then facilitate nitrogen use efficiency in plants. The results of this study provide a reference to applying this pattern in studying other intercropping systems.

## Results

### Cassava/peanut intercropping enhanced soil physicochemical properties

As shown in Table 1, after three continuous years planting cassava and peanut between March and July of each year, both in the monocropping and intercropping system, the soil physicochemical properties were significantly changed. The available N, P, K increased nearly 20-folds, and the organic matter increased by almost 40% compared to the control soils (*P* < 0.01). IP (IP, i.e., planting in peanut former cassava field) and IC (IC, i.e., planting cassava in former peanut field) cultivation patterns significantly promoted the soil nutrient contents, especially in available N (*P* < 0.01) and pH value.

**Table 1:** Basic soil physicochemical properties in the rhizospheric soils of MP, MC, IP and IC cultivation patterns

Treatments	Total N (g kg ^− 1^)	Total P (g kg ^− 1^)	Total K (g kg ^− 1^)	Available N (g kg ^− 1^)	Available P (g kg ^− 1^)	Available K (g kg ^− 1^)	Organic matter (g kg ^− 1^)	pH
MP	1.49 ± 0.017B	1.10 ± 0.017AB	6.05 ± 0.059A	0.124 ± 0.0012C	0.036 ± 0.0028A	0.277 ± 0.0017AB	23.13 ± 0.8647A	5.10 ± 0.1157B
MC	1.75 ± 0.015A	1.08 ± 0.045AB	5.95 ± 0.130A	0.126 ± 0.0003 BC	0.034 ± 0.0029A	0.208 ± 0.0015B	25.00 ± 1.249A	5.72 ± 0.1058A
IP	1.76 ± 0.055A	0.99 ± 0.020B	6.29 ± 0.064A	0.135 ± 0.0017A	0.027 ± 0.0015A	0.310 ± 0.0029A	25.77 ± 1.384A	6.12 ± 0.1099A
IC	1.77 ± 0.003A	1.18 ± 0.023A	6.01 ± 0.113A	0.131 ± 0.0013AB	0.036 ± 0.0058A	0.230 ± 0.0011AB	25.13 ± 0.982A	6.10 ± 0.0917A

Note: *MC* monocropping cassava, *MP* monocropping peanut, *IC* planting cassava in former peanut field, *IP* planting peanut in former cassava field

To figure out which enzymes play a crucial role in the intercropping system, five major soil enzyme activities were measured (Table 2). The catalase, sucrase, protease, and acid phosphatase activities in the rhizospheric soil of IC and IP cultivation patterns showed no difference with those in the monocropping cassava (MC) and monocropping peanut (MP) plants. However, urease activity was highest in IC, which was 78.5% more than that in MC. These results suggested that intercropping cassava in the peanut field significantly enhances urease activity and available N contents.

**Table 2:** The activities of five major enzymes in the rhizospheric soils of MP, MC, IP and IC cultivation patterns

Treatments	Urease activity (IU L^− 1^)	Protease activity (U L^− 1^)	Catalase activity (IU L^− 1^)	Sucrase activity (U L^− 1^)	Acid phosphatase activity (U L^− 1^)
MP	2.997 ± 0.0696B	14.416 ± 2.667A	17.742 ± 1.3336A	1.132 ± 0.1276A	1.540 ± 0.1157A
MC	2.278 ± 0.0606C	13.807 ± 2.018A	18.047 ± 1.0089A	1.203 ± 0.2498A	1.424 ± 0.0386A
IP	3.082 ± 0.0411B	12.921 ± 1.155A	18.489 ± 0.5776A	1.404 ± 0.1178A	1.920 ± 0.4649A
IC	4.067 ± 0.0312A	10.845 ± 1.159A	19.528 ± 0.5793A	1.381 ± 0.1715A	1.596 ± 0.0501A

Note: *MC* monocropping cassava, *MP* monocropping peanut, *IC* planting cassava in former peanut field, *IP* planting peanut in former cassava field

### Cassava/peanut intercropping increased the quantity of culturable microbial in the rhizospheric soil

Since the rhizospheric soil physicochemical properties changed after IC and IP cultivation, we tested the microbial quantity to characterize the relationship between microbes and soil physicochemical properties (Table 3). IP cultivation pattern induced a significant increase in the bacterial abundance, fungal abundance, and total microbial amount comparing with that in MC. In contrast, microbial Shannon-Wiener diversity index in the rhizospheric soil of IP was less than that of MC. These results indicated that intercropping peanut in cassava fields increased microbial quantity but decreased microbial diversity. IC significantly reduced the bacterial abundance, fungal abundance, and total microbial amount comparing with that of MP. However, the microbial Shannon-Wiener diversity index in the rhizospheric soil of IC was increased. These results indicated that intercropping cassava in peanut fields decreased microbial amount but increased microbial diversity.

**Table 3:** Microbial quantity in the rhizospheric soils of MP, MC, IP and IC cultivation patterns

Treatments	Bacteria (10^5^ g ^− 1^)	Fungi (10^2^ g ^− 1^)	Actinomyces (10^5^ g ^− 1^)	Total microbial population (10^5^ g ^− 1^)	Shannon-Wiener diversity index
MP	13.333 ± 0.6667A	36.417 ± 1.6915A	2.667 ± 0.4167A	16.036 ± 0.9451B	0.4615 ± 0.0284B
MC	5.333 ± 0.7265B	15.917 ± 1.3411B	3.500 ± 0.4330A	8.849 ± 1.0433C	0.6818 ± 0.0126A
IP	17.000 ± 1.4649A	37.333 ± 0.7949A	4.083 ± 0.2205A	21.121 ± 1.3025A	0.5042 ± 0.0299B
IC	5.917 ± 0.3005B	16.583 ± 0.6821B	2.750 ± 0.1443A	8.683 ± 0.1669C	0.6359 ± 0.0170A

Note: *MC* monocropping cassava, *MP* monocropping peanut, *IC* planting cassava in former peanut field, *IP* planting peanut in former cassava field

### Cassava/peanut intercropping changed the microbial community

**Fig. 1:**
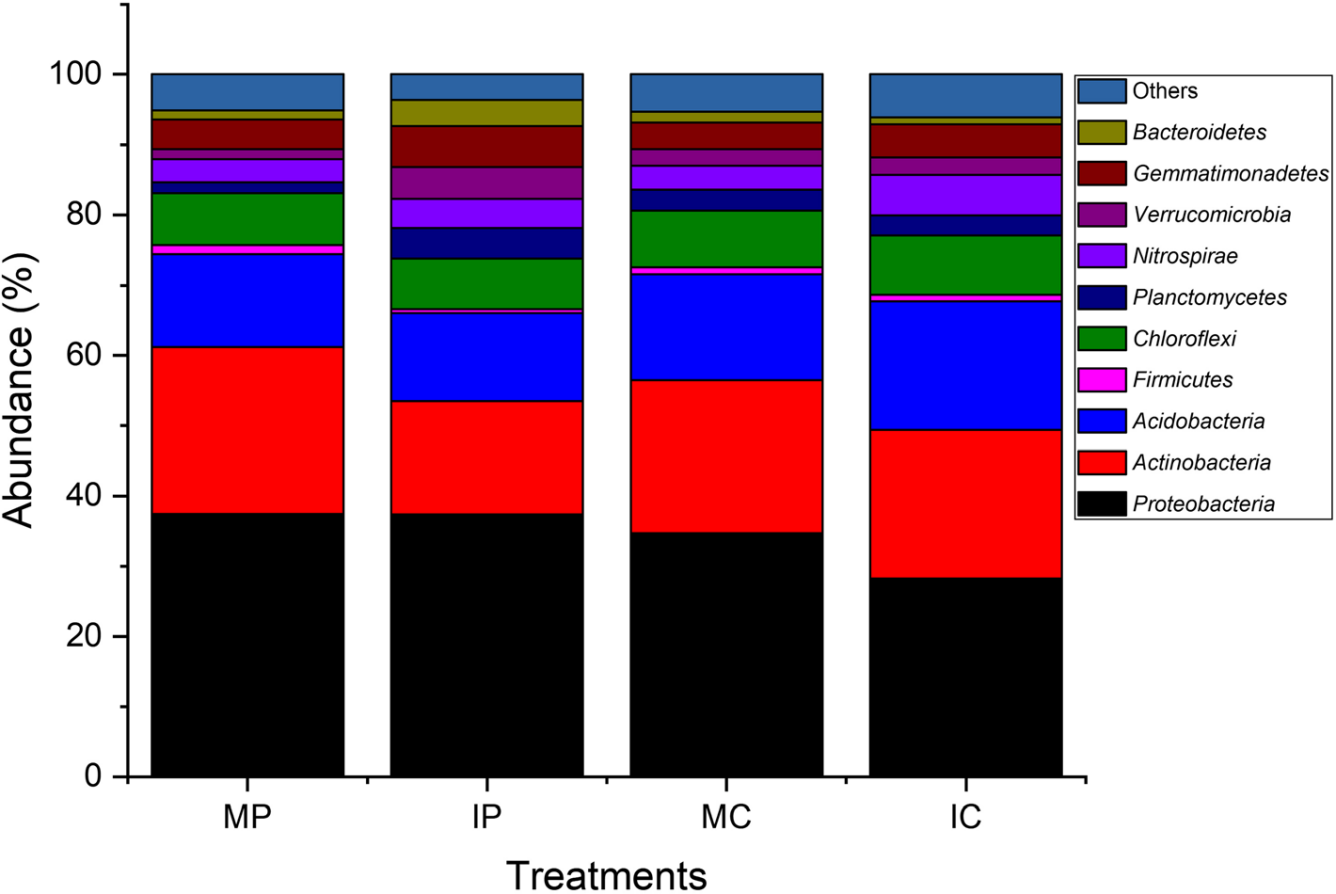
Taxonomic classification of bacterial reads in the rhizospheric soils of different cultivation patterns. MC, monocropping cassava; MP, monocropping peanut; IC, intercropping peanut/cassava; IP, intercropping cassava/peanut

**Fig. 2:**
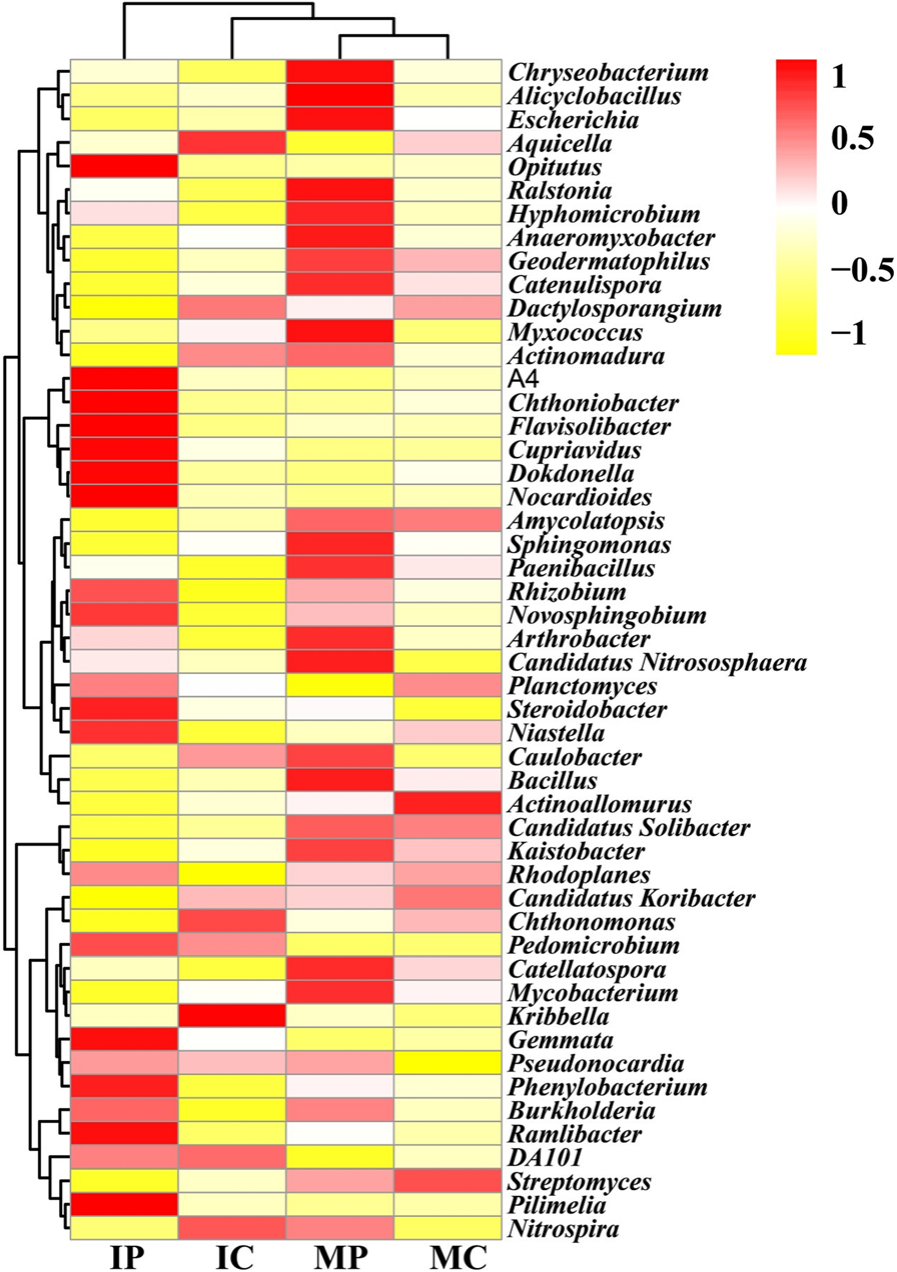
Heatmap analysis of the dominant genera in the rhizospheric soils of different cultivation patterns. MC, monocropping cassava; MP, monocropping peanut; IC, intercropping peanut/cassava; IP, intercropping cassava/peanut

To identify the change of microbial community in the intercropping systems, a total of 43,519 valid sequences were yielded by 16S rRNA gene sequencing using the Illumina MiSeq platform, which represented the vast diversity of the bacterial community (Figs. 1, 2). The taxonomic distribution at the phylum level is shown in Fig. 1. Proteobacteria was the most abundant phylum, accounting for 28.24 to 37.45% of the total valid reads in all the samples, with an average relative abundance of 34.45%. Actinobacteria was the second most abundant phylum, with an average relative abundance of 20.70%. The other dominant phyla were Acidobacteria (12.55–18.32%, with an average value of 14.79%), Chloroflexi (7.19–8.40%, with an average value of 7.76%), Gemmatimonadetes (3.80–5.83%, with an average value of 4.63%), *Nitrospirae* (3.27–5.81%, with an average value of 4.16%), Planctomycetes (1.60–4.36%, with an average value of 2.96%), Verrucomicrobia (1.46–4.51%, with an average value of 2.71%), and Bacteroidetes (1.03–3.75%, with an average value of 1.91%). Importantly, the percentages of Nitrospirae*,* Verrucomicrobia and Gemmatimonadetes in the rhizospheric soils of the IP and IC intercropping systems were more than those in the monocropping systems. Bacteroidetes and Planctomycetes were also more abundant in the rhizospheric soil of the intercropping system IP than those in the monocropping system MP. These phyla were also less abundant in MC than that in IC. Other phyla, such as Proteobacteria, Actinobacteria, Acidobacteria and Chloroflexi did not exhibit a significant difference between the monoculture and intercropping systems*.* On the genera levels (Fig. 2), four kinds of soils showed different dominant genera, for instance, *Aquicella, Chthonomonas, Kribbella, DA101* and *Nitrospira* were more abundant in the rhizospheric soil of the cassava plants in the IP and IC. *Actinoallomurus* and *Streptomyces* were still more abundant in the MC. Comparing with the rhizospheric soil of cassava, the rhizospheric soil of peanut exhibited higher diversity of dominant genera. There were 15 dominant genera in the rhizospheric soil of the intercropped peanut plants, including *Optitutus*, *A4*, *Chthoniobacter*, *Flavisolibater*, *Dokdonella*, and *Pilimelia*. However, these genera were not highly abundant in the peanut plants of monoculture system, which had different dominant genera, such as *Chryseobacterium, Alicyclobacillus, Escherichia, Ralstonia,* and *Hypomicrobium*. Thus, these results clearly demonstrated that the cassava/peanut intercropping changed the microbial community, which became distinct from that of the monocropping system. Furthermore, the microbial community in the rhizospheric soils of peanut and cassava plants is different due to different planting patterns, i.e. monocropping and intercropping systems.

### Principal component analysis (PCA) and redundancy analysis (RDA) of microbial communities and physicochemical properties in different cultivation patterns

**Fig. 3:**
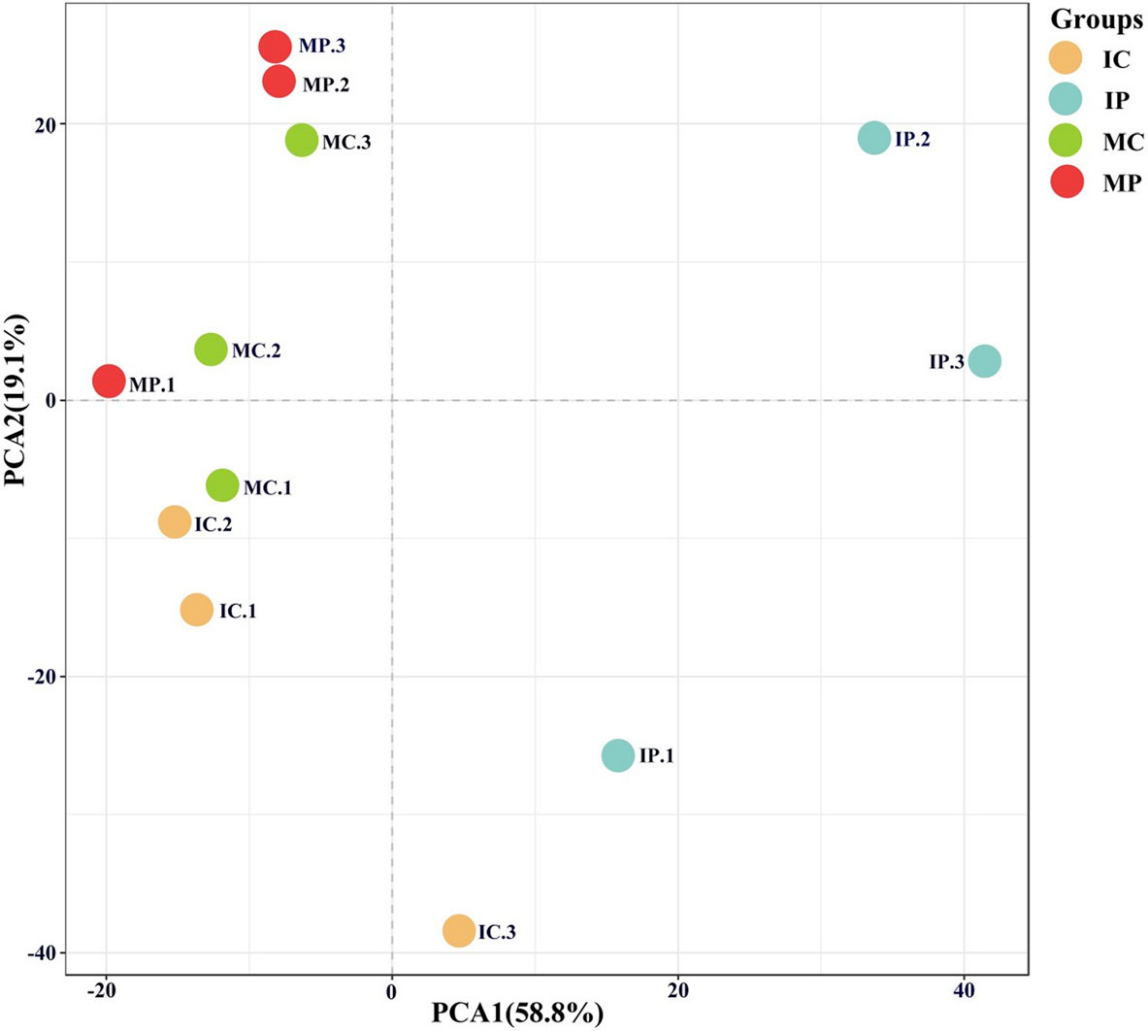
Principal component analyses of bacterial community in the rhizospheric soils of monocropping and intercropping systems based on Euclidean distance. MC, monocropping cassava; MP, monocropping peanut; IC, intercropping peanut/cassava; IP, intercropping cassava/peanut

**Fig. 4:**
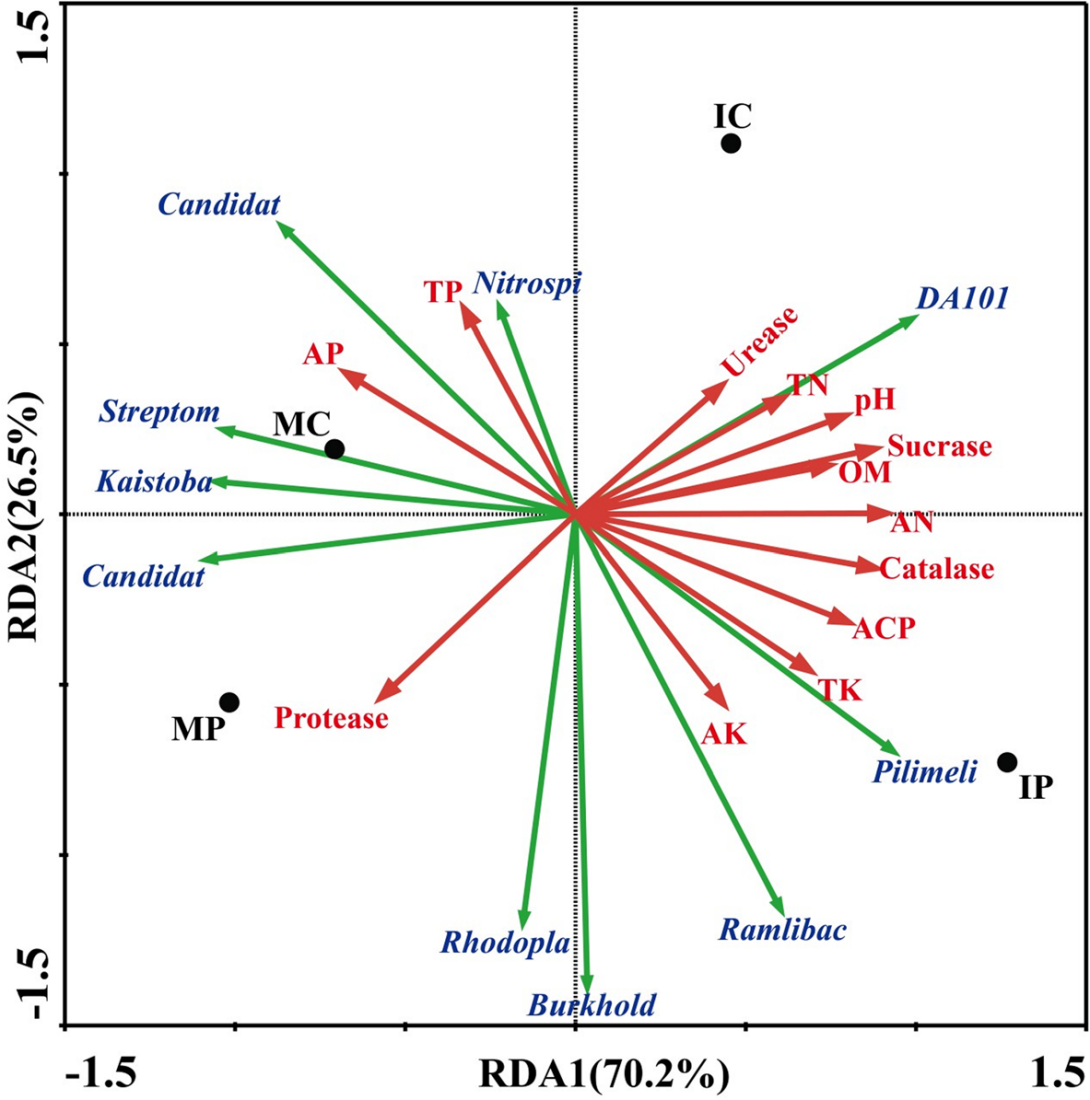
Redundancy analysis (RDA) of sequencing data of 16S rRNA gene and physicochemical properties in the rhizospheric soils of different cultivation patterns. MC, monocropping cassava; MP, monocropping peanut; IC, Intercropping peanut/cassava; IP, intercropping cassava/peanut

Principal component analysis showed that samples from monocropping and intercropping systems were separated from each other. The MP and IP were distributed in quadrant 2 and quadrant 1, 4, respectively (Fig. 3). It suggested that the same crop in different planting patterns influences the microbial communities accordingly. This finding may be associated with the environmental parameters in the different planting patterns. Microbial community exhibited a high correlation with intrinsic ecological parameters. Relationships between the important ecological parameters and the microbial community were discerned by RDA (Fig. 4). The length of the arrow corresponding to an ecological parameter indicated the strength of the ecological parameter concerning the overall microbial community. The results of RDA suggested that there were significant differences in the bacterial communities in the four planting patterns. As shown in Fig. 4, the available N, catalase, organic matter, sucrase activity, acid phosphatase activity, urease activity, total N, pH, total K, and available K were positively correlated with the RDA axis 1, and they were strongly and significantly associated with the overall microbial communities in IC and IP. In contrast, the total P, available P, and protease activity were negatively correlated with the RDA axis 1. These results revealed that available N, pH, catalase activity, and sucrase activity had the most significant impact on the microbial communities. Additionally, the abundances of some microbial genera, such as *DA101*, *Pilimelia*, and *Ramlibacter*, were positively correlated with available N, which were also the dominant genera in the soil of intercropped peanut plants (Fig. 1).

## Discussion

### Cassava/peanut intercropping system changed physicochemical properties of rhizospheric soils

Intercropping systems show great importance in agronomy, plant physiology, and ecology [41]. In general, intercropping systems change the bacterial diversity of soils [42], decrease disease rates [43, 44], and increase yields and cadmium accumulation [45]. In this study, rhizospheric soils both in IP and IC systems showed higher available N and pH than those in MP and MC systems, respectively. These were consistent with previous studies. For instance, intercropping of green garlic (*Allium sativum* L.) with cucumber (*Cucumis sativus* L.) increased organic matter and available N, P and K contents in soils [46]. Eggplant/garlic relay intercropping system increased available N contents from 61.95 mg kg^− 1^ to 76.30 mg kg^− 1^ [47]. In sugarcane-soybean intercropping system, available N increased by 10% [29]. The increase of available N was related to the urease activities, which was the only significant change in IC and IP comparing with that in MC and MP (Table 2). The role of urease is to catalyze urea hydrolysis into ammonia and carbon dioxide [48, 49], which is common in higher plants, bacteria, fungi, and algae. Thus, we speculate that the changes of soil physicochemical properties may be related to urease produced by microbial community since both the dominant genera and quantity of the microbial community were changed in IC and IP comparing with that in MC and MP (Table 3).

### Improvement of physicochemical properties of rhizospheric soils was related to the microbial community in cassava/peanut intercropping system

**Fig. 5:**
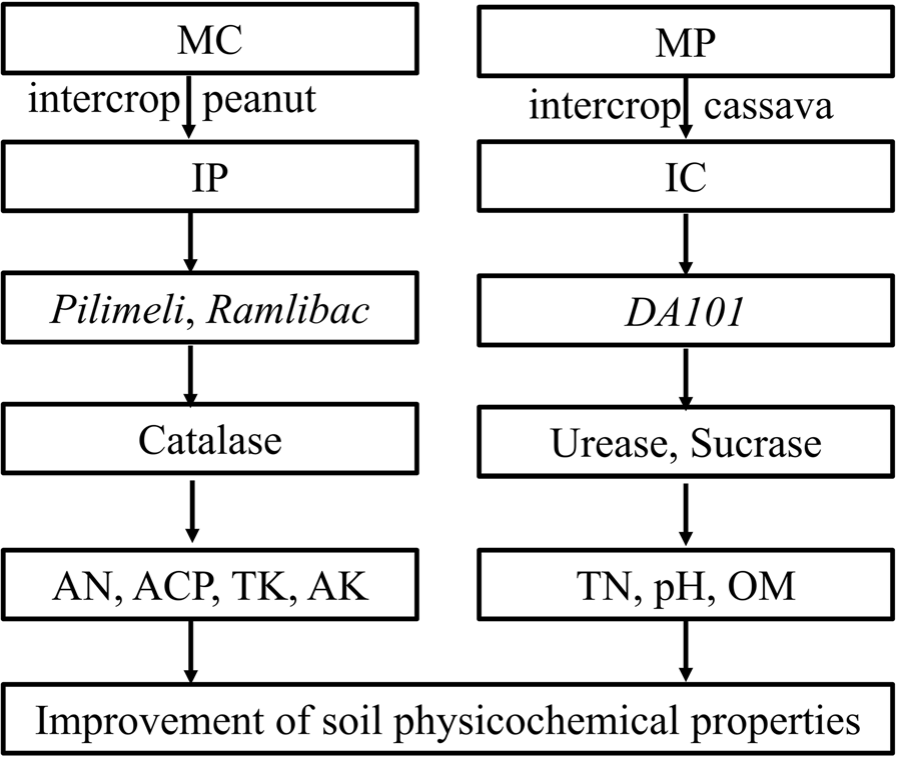
Models of IP and IC enhanced soil physicochemical properties. MC, monocropping cassava; MP, monocropping peanut; IC, peanut/cassava intercropping; IP, cassava/peanut intercropping

Through MiSeq sequencing analysis of the 16S rRNA gene, we found that the percentage of Nitrospirae, Verrucomicrobia, and Gemmatimonadetes in the rhizospheric soils of IP and IC intercropping systems were higher than those in the monocropping systems (Fig. 1). This finding is quite different from the result of a previous study using a different detection technique [50]. In the previous study, denaturing gradient gel electrophoresis (DGGE) was used to analyze microbial community structure and diversity, and only ten kinds of fungi and bacteria were identified [50]. In this study, the new MiSeq sequencing technique was used, and a total of 50 species were identified (Fig. 3). Proteobacteria and Actinobacteria were the most abundances bacteria in different intercropping systems [49], while other species were varying. For instance, Firmicutes was the dominant genus in mulberry/alfalfa intercropping soils [49], but it accounted for only 0.54–1.33% in this study (Fig. 1). These results suggested that each intercropping system had different mechanisms, especially in the composition of bacterial community. Based on genus analysis, PCA and RDA analysis, it was clear that the differences in the IC and IP systems comparing with those in MC and MP systems (Figs. 2, 3 and 4) were related to the abundance of *DA101*, *Pilimeli*, and *Ramlibac*. Then these bacteria increased enzyme activities and improved the physicochemical properties of rhizospheric soil (Fig. 5). Unclassified *DA101* belongs to the family Chthoniobacteraceae, phylum Verrucomicrobia, which was found in the soils of pepper tree [51], and grassland [52]. The abundance of *DA101* was increased in the soil of intercropping system, but it was negatively correlated with the total N content and pH at significant level [53]. In this study, we found that *DA101* abundance was significantly and positively correlated with the available N content in cassava/peanut intercropping system, which was consistent with the change in the abundance of the phylum Verrucomicrobia in the intercropping system (Fig. 2-4). These results indicated that the available N content, and not the total N content was related to *DA101* abundance. Recently, researchers assembled a draft genome of *Candidatus Udaeobacter copiosus*, which is a representative of the *DA101* clade, and speculated that this organism is a soil oligotrophic bacterium which reduces its requirement for soil organic carbon by acquiring amino acids and vitamins from the environment [54]. The functions of *DA101* need to be investigated more deeply. *Ramlibacter tataouinensis* TTB310^T^ (strain TTB310) is a beta-proteobacterium isolated from sand particles [55]. Different species from this genus were isolated in tropical forest soil [56] and the rhizospheric soil of *Mugunghwa* [57]. Furthermore, studies revealed that the genus of Ramlibacter has phosphatase activity [58] and ginsenoside-converting activity [59]. The relationship between these bacteria and enzymes activities should be studied further to reveal their functions.

## Conclusions

The inner mechanism of cassava/peanut intercropping system was elucidated through analyzing the physicochemical properties of rhizospheric soils and microbial community. We found that the cassava/peanut intercropping system increased the quantities of *DA101*, *Pilimelia*, and *Pilimelia*, and thereby increased the available N content in the soil and improved soil quality.

## Methods

### Cassava and peanut plants

Cassava variety Huanan 205 and shade-tolerant peanut variety Guihua 836 were provided by the Cash Crops Research Institute of the Guangxi Academy of Agricultural Sciences. All cassava and peanut experiments were performed in the Lijian Scientific Base, which is certified for the field cultivation experiment by local government.

### Experimental site and soil

The experiments were performed in the Lijian Scientific Base (23°14′25′′N, 108°03′42′′E) of the Guangxi Academy of Agricultural Sciences, Nanning City, Guangxi province, China. The field site was previously used for monocropping cassava and peanut. The tested soil was acid red loam, with total nitrogen content (total N), total phosphorus content (total P), total potassium content (total K), available nitrogen content (available N), available phosphorus content (available P), available potassium content (available K), the organic matter content and pH value of 1.34 g kg^− 1^, 0.53 g kg ^− 1^, 12.6 g kg ^− 1^,0.0705 g kg ^− 1^, 0.0139 g kg ^− 1^, 0.098 g kg ^− 1^, 16.2 g kg ^− 1^, and 5.8, respectively.

### Experimental design and management

**Fig. 6:**
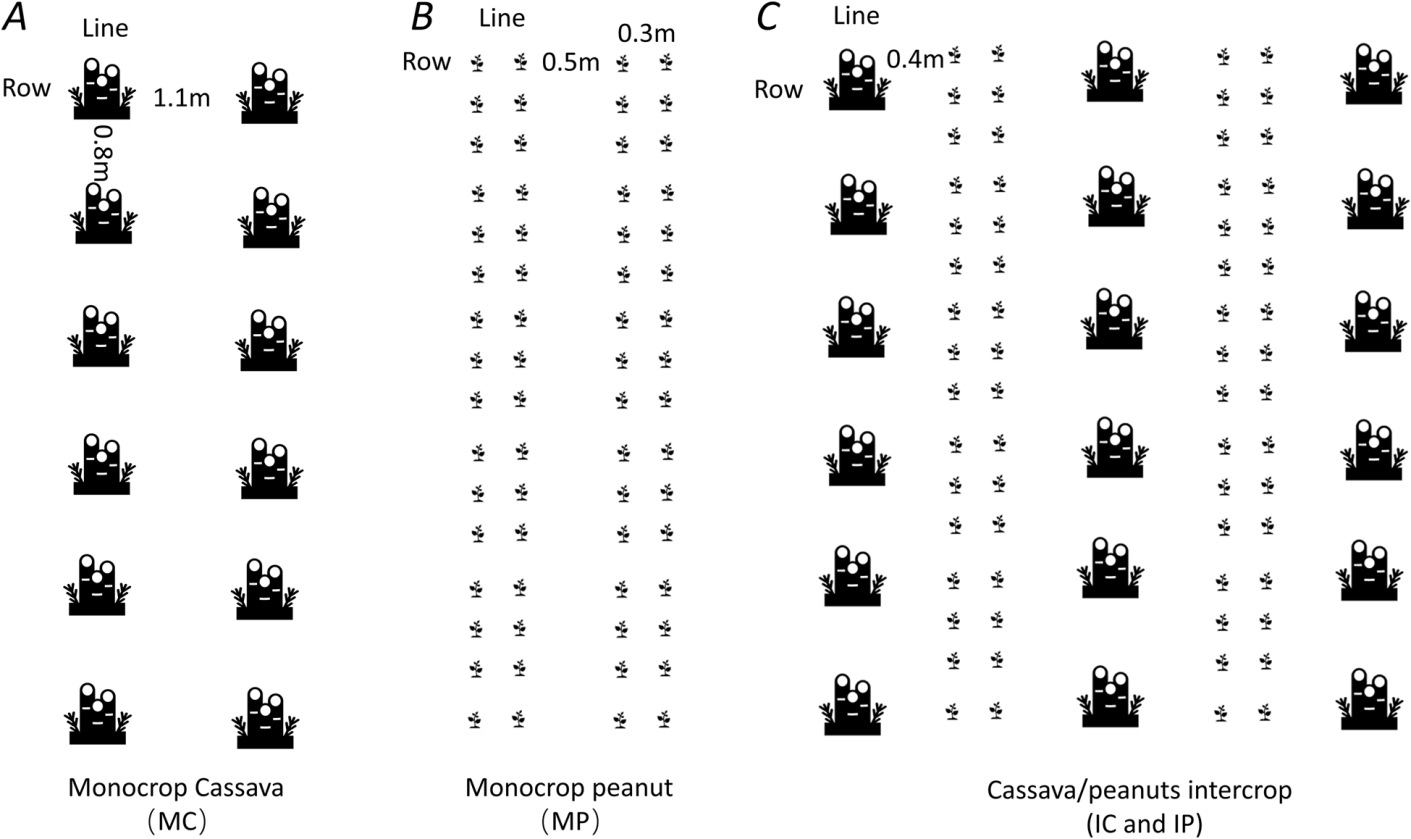
Diagram of experimental design

In March 2016–2018, cassava and peanut were planted simultaneously in the field. Monocropping cassava (MC), monocropping peanut (MP) crops were compared with peanut/cassava intercropping (IC, i.e., planting cassava in former peanut field) and cassava/peanut intercropping (IP, i.e., planting peanut in former cassava field) (Fig. 6). For MC, cassava planted with a row spacing of 1.1 m × 0.8 m and with equivalent line spacing. For MP, the peanut planted in a narrow-wide row spacing pattern. The line spacing for peanut in the wide line was 0.5 m, and the row spacing in the narrow row was 0.3 m × 0.16 m. For IC and IP, two lines of peanut were planted alongside one line of cassava. The line spacing between cassava and peanut was 0.4 m. The row spacing for cassava and peanut intercropping was 1.1 m × 0.8 m and 0.3 m × 0.16 m, respectively. The experiment was arranged in plots (6 m × 8 m) in a randomized design with three replicates in each treatment.

Peanut was supplied with 450 kg ha^− 1^ compound granulated NPK fertilizers (N-P_2_O_5_-K_2_O = 15–15-15) and 750 kg. ha^− 1^ fused calcium-magnesium phosphate fertilizer (available P_2_O_5_ 18%). Cassava was supplied with 750 kg Ha^− 1^ compound granulated NPK fertilizers. The crops were irrigated two times during crop growth period based on crop water requirement and soil water content. Pesticide and herbicide were applied about two months after sowing.

### Soil sampling

In July 2016–2018, the time of harvesting mature peanut, ten plants of cassava and peanut per treatments were uprooted. The rhizospheric soil from both loose soil and cohesive soil from the plant roots were collected, mixed, and separated into three sealed virus-free bags for the following assays. One sample was maintained in the refrigerator at 4 °C and used for determining the culturable soil microbe. One sample was maintained in the refrigerator at − 80 °C and used for extracting soil DNA and high-throughput sequencing. The last sample was dried naturally, grounded, and sieved for determining the soil physicochemical properties.

### Soil physicochemical property analysis

The physicochemical properties were measured according to the previous reports [49]. The available N contents were measured by the alkaline hydrolysis diffusion method. The available P was measured by sodium bicarbonate extraction/Mo-Sb colorimetry method. The available K was measured by ammonium acetate extraction/flame photometry method. Organic matter content was measured by the potassium bichromate titrimetric method. Soil enzyme activities were measured according to the previous reports. Catalase activity [60], sucrase activity, proteinase activity, urease activity [48], and acid phosphatase activity [3] were measured by permanganate titration, sodium thiosulfate titration, ninhydrin colorimetry, indophenol blue colorimetry, and the disodium phosphate benzene colorimetric method, respectively.

### Determination of soil microbial quantity

Soil microbial quantity was measured by the conventional microculture method. Bacteria, fungi, and actinomycetes were cultured in beef extract-peptone medium, Martin medium, and Gao 1 medium, respectively. The Shannon-Wiener index was used to calculate the biodiversity index (H): H = −Σ(ni/N) × ln(ni/N). In this formula, ni is the microbial quantity of species i, and N is the total microbial quantity [61].

### Soil DNA extraction, PCR amplification, high-throughput sequencing and analysis

Total genomic DNA was extracted from the samples using the FastDNA SPIN Kit (MP Biomedicals, Santa Ana, USA) according to the manufacturer’s instructions. The DNA concentration and purity were monitored on 1% agarose gels, and the DNA was diluted to 1 ng/μL using sterile water. The DNA was stored at − 80 °C until subsequent PCR amplification. The total genomic DNA was subjected to PCR amplification using the primer pair 515f/806r, which amplified the V4 region of the 16S rDNA gene [62], following a previously described protocol [63]. PCR was carried out in 30-μL reactions with 15 μL of Phusion® high-fidelity PCR master mix, 0.2 μM of forward and reverse primers, and approximately 10 ng of template DNA. Thermal cycling consisted of an initial denaturation at 98 °C for 1 min; followed by 30 cycles of denaturation at 98 °C for 10 s, annealing at 50 °C for 30 s, and elongation at 72 °C for 30 s; and a final step at 72 °C for 5 min. The PCR products were mixed with equal volumes of 1× loading buffer (containing SYBR Green), and electrophoresis conducted on a 2% agarose gel for detection. Samples with a bright band at 400–450 bp were chosen for further experiments. The PCR products were mixed at equal concentrations. Then, the mixture of PCR products were purified with the GeneJET Gel Extraction Kit (Thermo Scientific). Sequencing libraries were generated using the NEB Next® Ultra™ DNA Library Prep Kit for Illumina (NEB, USA) following the manufacturer’s recommendations, and the index codes were added. The library quality was assessed on a Qubit 2.0 fluorometer (Thermo Scientific) and an Agilent 2100 bioanalyzer system. Finally, the library was sequenced on an Illumina MiSeq platform by the Novogene Corporation (Beijing, China), and 250/300-bp paired-end reads were generated.

### Illumina MiSeq sequencing analysis

Paired-reads from the original DNA fragments were merged based on a previously described method [64]. Sequencing reads were assigned to each sample according to the individual unique barcodes, then analyzed with the QIIME (Quantitative Insights Into Microbial Ecology) software package and the UPARSE pipeline [65]. The reads were first filtered by QIIME quality filters. Default settings for Illumina processing in QIIME were used. Then, the UPARSE pipeline was used to select operational taxonomic units (OTUs) with 97% similarity. For each OTU, a representative sequence was selected and used to assign taxonomic composition by the RDP classifier. Then, the estimated species richness was determined by rarefaction analysis [66]. Redundancy analysis (RDA) was performed to analyze the correlation between environmental factors and microbial community.

### Data analysis

Principal component analysis (PCA) was performed with SIMCA software v.13.0 (Umetrics, Sweden) [67]. The means and standard errors of three repeats in MC, MP, IC, and IP planting patterns were analyzed by One-way variance analysis with SPSS24.0, and Duncan’s test of the homogeneity of variance was performed with the confidence level of 0.01 [68].

## Data Availability

Raw data of 16S rRNA gene obtained from all samples are accessible via NCBI SRA database under accession number PRJNA606845.

## Abbreviations

IC: Intercropping peanut/cassava
IP: Intercropping cassava/peanut
MC: Monocropping cassava
MP: Monocropping peanut
OTUs: Operational taxonomic units
PCA: Principal component analysis
RDA: Redundancy analysis
